# A new species of *Thecadactylus* from Sint Maarten, Lesser Antilles (Reptilia, Squamata, Gekkonidae)

**DOI:** 10.3897/zookeys.118.1476

**Published:** 2011-07-13

**Authors:** Gunther Köhler, Milan Vesely

**Affiliations:** 1 Forschungsinstitut und Naturmuseum Senckenberg, Senckenberganlage 25, 60325 Frankfurt a.M., Germany; 2 Department of Zoology, Faculty of Natural Sciences, Palacký University, tŕ. Svobody 26, 77146 Olomouc, Czech Republic

**Keywords:** Gekkonidae, Lesser Antilles, new species, Reptilia, Sint Maarten, Squamata, *Thecadactylus*

## Abstract

We describe a new species of *Thecadactylus*from the Caribbean island of Sint Maarten. The new species differs from all other species in the genus by having a distinct dorsal pattern of numerous irregular but sharply deliminated black spots and blotches on an otherwise almost patternless background.

## Introduction

Turnip-tailed geckos (genus *Thecadactylus*) are moderate-sized to large geckos distributed from southeastern Mexico across most of Central America and mesic tropical South America, and also occupy the Lesser Antilles (Russell and Bauer 2002). Whereas these geckos have traditionally been understood as a monotypic genus (e.g., Peters and Donoso-Barros 1970; Hoogmoed 1973; Avila-Pires 1995), currently two species of *Thecadactylus* are recognized (Bergmann and Russell 2007): *Thecadactylus rapicauda* (Houttuyn, 1782) and *Thecadactylus solimoensis* Bergmann & Russell, 2007. All *Thecadactylus* populations from the Lesser Antilles have always been considered as conspecific with *Thecadactylus rapicauda* (Powell et al. 1996, Censky and Kaiser 1999, Malhotra and Thorphe 1999).

In 2010, several specimens of a distinctly spotted *Thecadactylus* were collected on the island of Sint Maarten (also known as Saint Martin), Lesser Antilles, and imported to Germany by a pet trade dealer. In order to verify the geographic origin of these individuals, Stephan Prein and Maciej Oskroba visited Sint Maarten in April 2011. They indeed encountered *Thecadactylus* there and found that all specimens from this island had a distinctly spotted but otherwise patternless dorsum. A research of the pertinent literature revealed that this peculiar form had already been reported and illustrated from Sint Maarten (Breuil 2002, 2003). A comparison with the *Thecadactylus* from many localities across the wide geographic range of these geckos demonstrated that the Sint Maarten population represents an undescribed species and therefore, we describe it as a new species below.

## Materials and methods

A list of the comparative specimens examined is provided in the Appendix. Abbreviations for museum collections follow those of Leviton et al. (1985). Furthermore, we have studied and analysed photographic material published in Powell (1996), Breuil (2002), Russell and Bauer (2002), and Powell et al. (2005). Nomenclature of scale characters follows that of Avila-Pires (1995). Subdigital lamellae were counted as suggested by Bergmann and Russell (2003). Scale sizes were measured using the ocular micrometer of a stereo microscope (Leica MZ 12) and rounded to the nearest 0.01 mm. All other measurements were made using precision calipers and were rounded to the nearest 0.1 mm. Head length was measured from the tip of the snout to the anterior margin of the ear opening. Snout length was measured from the tip of the snout to the anterior border of the orbit. Head width was determined as the distance between the oral ricti. Tail height and width were measured at the point reached by the heel of the extended hind leg. Dorsal and ventral scales were counted at midbody along the midline. Abbreviations used are DHL (number of medial dorsal scales in one head length), HL (head length), HW (head width), INL (infralabials), SAM (scales around midbody), SPL (supralabial scales), SVL (snout–vent length), and VHL (number of medial ventral scales in one head length). For the synonymy list, only those works have been included that cite actual specimens from Sint Maarten. Temperature was recorded at the type locality (see below) in the time from 11 – 16 April 2011 using an automatic temperature data logger (HOBO Pendant temp) placed on an upstanding tree trunk about 3 m above the ground in the shade recording at intervals of 2 min.

## Results

### Thecadactylus oskrobapreinorum sp. n.

urn:lsid:zoobank.org:act:0F9770AC-C296-462A-B23F-F3356ECC4BE5

http://species-id.net/wiki/Thecadactylus_oskrobapreinorum

Figs 1 [Fig F2] –3

Thecadactylus rapicauda: (Breuil (2002, 2003; in part.), Bergmann and Russell (2007; in part.).

#### Holotype.

SMF 92120, an adult male from Sint Maarten, near the southern edge of the village of Dawn Beach, 18.042°N, 63.023°W, 45 m elevation; collected 12 April 2011 by Stephan Prein and Maciej Oskroba.

#### Paratypes.

SMF 92194, 92721–29, same collecting data as holotype.

#### Diagnosis.

A species (SVL in largest specimen examined 99 mm) of the genus *Thecadactylus*(sensu Russell and Bauer 2002) that differs from all other species in the genus by having a distinct dorsal pattern of numerous irregular but sharply deliminated black spots and blotches on an otherwise almost patternless background. *Thecadactylus oskrobapreinorum* lacks a dorsally directed postocular stripe (such stripe present in most specimens of *Thecadactylus solimoensis*; see Bergmann and Russell 2007). *Thecadactylus oskrobapreinorum* differs further from *Thecadactylus rapicauda* in the mean values of several morphometric and pholidotic characteristics, most pronounced in the number of subdigital lamellae and supralabial scales (see Table 1).

#### Description of the holotype.

Adult male as indicated by partially everted hemipenes; SVL 95.5 mm; tail length 75.0 mm, tail complete; tail almost round in cross section, tail height 6.9 mm, width 7.6 mm; axilla to groin distance 37.0 mm; head length 24.5 mm; snout length 12.2 mm; head width 20.5 mm; shank length 16.2 mm. Rostral large, rectangular, about twice as wide as deep, visible from above, and with a long median cleft extending from posterior margin; 2 relatively large, rectangular postrostrals; nostril bordered by rostral, first supralabial, 3 small postnasals and one postrostral; scales on snout and on loreal region granular, mostly keeled; 22 loreal scales in a longitudinal line between rostral and orbit; scales on upper and posterior portions of head slightly smaller than on snout; scales in supraorbital region not differentiated from those on upper part of head; supraciliary flap bordered by a double row of scales, 18 in outer row between anterior border of flap and a point above center of eye, with 7 small spines posteriorly; pupil four-lobed, vertically elongate; 8 supralabials to level below center of eye, total number 10, anterior supralabials subequal in size, below eyes decreasing in size; ear opening obliquely oval, 3.0 x 1.5 mm (length x height) distinctly smaller than eye (eye length 6.1 mm); mental larger than adjacent scales, pentagonal; 2 relatively large postmentals, at each side followed by a row of smooth, polygonal scales, decreasing in size posteriorly, and in contact with anterior infralabials; scales on chin granular, mostly pointed; scales on throat small, round, convex, juxtaposed; 8 (right)–7 (left) infralabials to level below center of eye, total number 10; infralabials mostly large, smooth, quadrangular to pentagonal, posterior ones smaller; dorsum of body with convex, juxtaposed to subimbricate scales with rounded posterior margins, about twice as large as scales on snout, largest dorsal scales about 0.35 x 0.29 mm (length x width); about 72 median dorsal scales in one head length; ventral scales at midbody smooth, juxtaposed to subimbricate with rounded posterior margins, forming oblique rows, about 0.42 x 0.39 mm (length x width); about 45 ventral scales in one head length; a gradual transition between dorsal and ventral scales; 218 scales around midbody; caudal scales smooth, imbricate, with rounded posterior margins, slightly larger ventrally; scales on limbs mostly smooth, subimbricate, with rounded posterior margins, equal to, to larger than dorsals; scales on posterior surfaces of forelimbs and on posterior and upper surfaces of hind limbs small, granular; fingers and toes depressed with a middorsal elevation, connected by a basal web; subdigital lamellae forming two transversely enlarged rows, divided by a median sulcus, 20 under fourth toe, 19 under fourth finger; claw on distal extremity of distal sulcus.

Coloration after one month in preservative (70% ethanol) was recorded as follows: Dorsal surfaces of head, body, limbs, and tail grayish brown with numerous irregular but sharply deliminated, black spots and blotches; ventral surfaces of head, body, and limbs cream with gray shading and faint gray reticulations; widened lamellae of fingers and toes gray; ventral surface of tail brown with dark grayish brown reticulations.

#### Variation.

The paratypes agree well with the holotype in general appearance, morphometrics and scalation (see Table 1). Variation of coloration in life is illustrated in Fig. 4. As can be seen, the number and distribution of the dark spots and blotches varies between individuals as does the background color which ranges from pale pearl gray over pale grayish yellow to grayish olive. Scale size differences in certain areas of gular and temporal region of male holotype are probably the result of bites from other males in territorial fights. The damaged parts of the skin are covered by smaller granular scales as it is typical for scar tissue.

#### Etymology.

The name *oskrobapreinorum* is a construction in the genitive plural honoring Maciej Oskroba and Stephan Prein, two German herpetoculturists who directed our attention to this new species and made field observations on this gecko on the island of Sint Maarten.

#### Natural history notes.

All type specimens were collected at night while the lizards were active on the lower parts of the trunks of large living trees within or at the edge of forested areas (see also Fig. 5). From 11–16 April 2011, the air temperature (measured in the shade) varied at the type locality from 21.1–23.7°C (mean 22.7°C) in the morning hours and 24.6–28.2°C (mean 26.6°C) in the afternoon.

#### Geographic Distribution.

As currently known, *Thecadactylus oskrobapreinorum* is restricted to the island of Sint Maarten, Lesser Antilles. Although the type locality is in the Dutch portion of the island, the species is also known from several localities in the French portion (see records in Breuil 2002).

## Discussion

The distinctive dorsal pattern in *Thecadactylus oskrobapreinorum* appears to be a fixed character in this species since no individuals without dark spots on a otherwise patternless dorsum have been documented (see also a photo of a specimen of this species in Breuil 2002, 2003). As described and illustrated, individuals of the *Thecadactylus* populations from the nearby islands of St. Eustatius, St. Barthelemy, and Saba show the “normal” dorsal pattern of *Thecadactylus rapicauda* (Breuil 2002, Powell et al. 2005). However, Breuil (2002) pictures a specimen from the island of La Désiderade which exhibits a strikingly aberrant coloration with a almost white head contrasting with the mostly dark grayish body, limbs, and tail. A more comprehensive survey and analysis of variation, both of molecular genetic and of morphological traits, is needed in order to shed light on the actual species diversity of *Thecadactylus* on the Lesser Antilles.

We have not examined the types of the nominal species placed in the synonymy of *Thecadactylus rapicauda* (following e.g., Peters and Donoso-Barros 1970, Russell and Bauer 2002). However, based on the respective type localities, even given the vague nature of most of them, none of them came from near Sint Maarten, not even from the Lesser Antilles: *Gekko laevis* Daudin, 1802 (type locality: “Amérique méridionale”); *Gekko surinamensis* Daudin, 1802 (type locality: “Surinam”); and *Pachydactylus tristis* Hallowell, 1854 (type locality: “Liberia, west coast of Africa”, in error fide Russell and Bauer 2002). Therefore, none of the aforementioned names can be applied to the species described herein. In the cases of *Gekko laevis* and *Pachydactylus tristis*, the types of both of which are lost and no accurate type locality given, the synonymy assignment to either *Thecadactylus rapicauda* or *Thecadactylus solimoensis* remains unsettled. Also, as pointed out by Bergmann and Russell (2007), additional molecular genetic work with more intensive sampling is needed in order to clarify the geographic boundaries between *Thecadactylus rapicauda* and *Thecadactylus solimoensis*.

**Figure 1.**
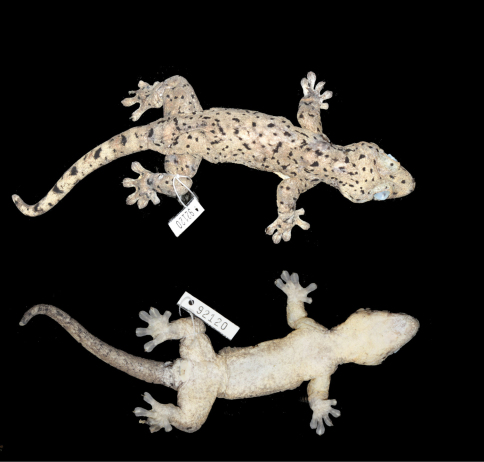
Holotype of *Thecadactylus oskrobapreinorum* (SMF 92120). SVL = 95.5 mm.

**Figure 2. F2:**
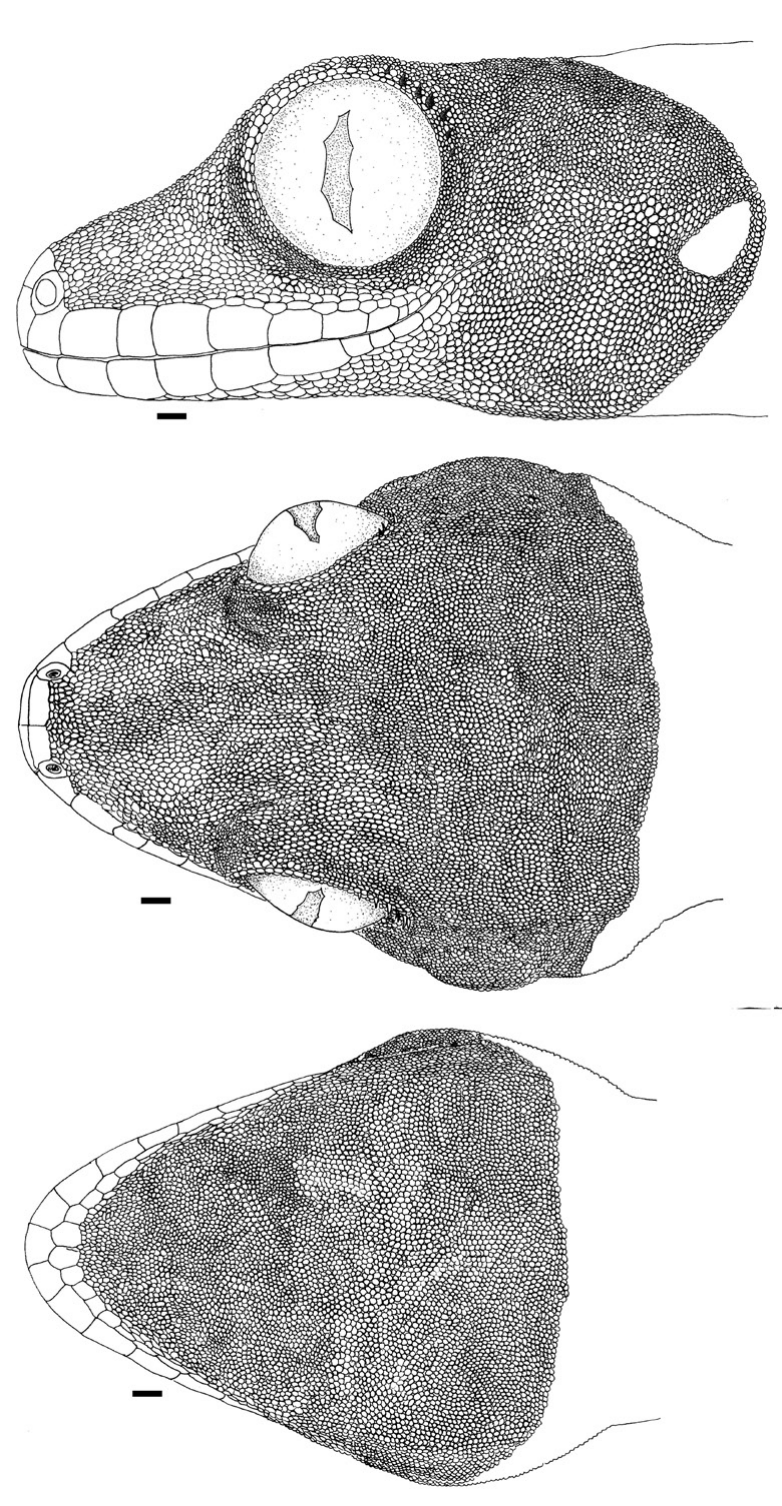
Head of holotype of *Thecadactylus oskrobapreinorum* (SMF 92120). Scale bar equals 1.0 mm.

**Figure 3.**
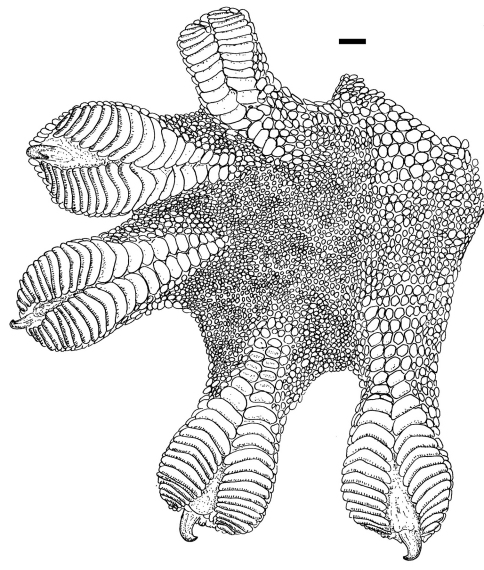
Right hind foot of holotype of *Thecadactylus oskrobapreinorum* (SMF 92120). Scale bar equals 1.0 mm.

**Figure 4.**
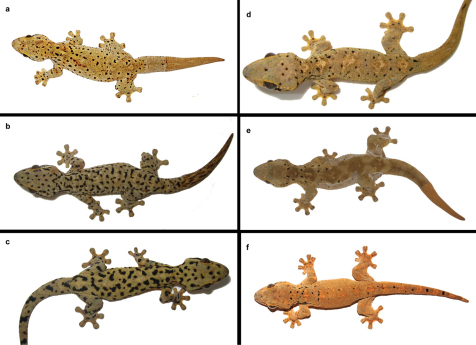
*Thecadactylus oskrobapreinorum* in life (specimens not preserved; all from Sint Maarten, Lesser Antilles). Photos a,e by Gunther Köhler; b,c,d by Stephan Prein, f by Maciej Oskroba

**Figure 5.**
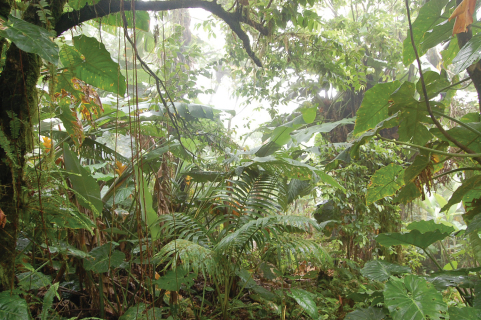
Habitat at the type locality of *Thecadactylus oskrobapreinorum* on Sint Maarten, Lesser Antilles. Photo by Maciej Oskroba

**Table 1.** Selected measurements, proportions and scale characters of *Thecadactylus oskrobapreinorum* and *Thecadactylus rapicauda*. Range is followed by mean value and standard deviation in parentheses. For abbreviations see text. For tail length, only complete original tails were measured.

		*Thecadactylus oskrobapreinorum*♂ 4♀ 4	*Thecadactylus rapicauda*♂ 11♀ 9
SVL	♂	86.0–96.5 (90.6 ± 3.83)	74.0–95.0 (85.5 ± 6.87)
	♀	87.0–99.0 (94.0 ± 4.85)	79.0–119.0 (93.9 ± 11.65)
Tail length	♂	75.0–76.0 (75.3 ± 0.47)	65.0–72.0 (68.5 ± 3.50)
	♀	71.0–83.0 (77.0 ± 6.00)	75.0–76.0 (75.3 ± 0.47)
HL	♂	22.6–24.5 (23.5 ± 0.67)	18.2–24.7 (21.5 ± 1.75)
	♀	23.0–25.0 (24.3 ± 0.79)	18.8–28.0 (23.2 ± 2.56)
HW	♂	17.3–20.5 (18.4 ± 1.25)	14.7–19.9 (17.3 ± 1.95)
	♀	17.6–19.1 (18.2 ± 0.58)	13.2–22.9 (17.9 ± 2.74)
Shank length	♂	13.2–16.2 (14.2 ± 1.21)	10.0–14.5 (12.7 ± 1.29)
	♀	14.0–15.9 (14.5 ± 0.81)	10.6–17.2 (14.3 ± 1.95)
Axilla–groin distance	♂	37.0–38.7 (37.6 ± 0.68)	33.0–45.7 (39.1 ± 4.54)
	♀	32.5–43.0 (39.2 ± 4.14)	37.7–49.5 (42.5 ± 3.83)
Tail length / SVL	♂	0.78–0.87 (0.83 ± 0.04)	0.82–0.87 (0.85 ± 0.02)
	♀	0.72–0.90 (0.81 ± 0.09)	0.78–0.87 (0.83 ± 0.04)
HL / SVL	♂	0.25–0.27 (0.26 ± 0.01)	0.24–0.26 (0.25 ± 0.01)
	♀	0.25–0.27 (0.26 ± 0.01)	0.24–0.27 (0.25 ± 0.01)
Shank length / SVL	♂	0.15–0.17 (0.16 ± 0.01)	0.13–0.17 (0.15 ± 0.01)
	♀	0.14–0.16 (0.15 ± 0.01)	0.13–0.17 (0.15 ± 0.01)
Axilla–groin distance / SVL	♂	0.38–0.43 (0.42 ± 0.02)	0.41–0.53 (0.46 ± 0.03)
	♀	0.37–0.43 (0.42 ± 0.02)	0.42–0.49 (0.46 ± 0.02)
Subdigital lamellae of 4th toe		16–20 (18.13 ± 1.17)	18–23 (20.17 ± 1.62)
Subdigital lamellae of 4th finger		16–19 (17.88 ± 1.05)	17–23 (19.67 ± 2.13)
Number of SPL to level below center of eye		6–8 (6.63 ± 0.70)	8–10 (8.62 ± 0.62)
Number of INL to level below center of eye		8–10 (8.63 ± 0.70)	7–8 (7.62 ± 0.49)
Number of postrostrals		2	2
Number of postmentals		2	2
Number of medial dorsal scales in one head length		72–92 (81.25 ± 7.60)	64–88 (75.15 ± 8.24)
Number of medial ventral scales in one head length		44–56 (49.00 ± 4.24)	34–52 (40.62 ± 4.68)

## Supplementary Material

XML Treatment for Thecadactylus oskrobapreinorum

## Appendix

### Comparative material examined

*Thecadactylus rapicauda* – **Brazil:** Maranhão: SMF 8378. **Colombia:** Melgar, Tolima: SMF 70233. **Guatemala:** Sitio Arqueologico Quirigua, 76 m: SMF 84018. **Honduras:** Isla de Utila, near Iguana Station: SMF 79871; Isla de Utila, 1 km S Rock Harbour: SMF 77098; west end of Isla de Utila: SMF 77099; Río Platano Biosphere Reserve, vicinity of Río Cuyamel, 15°34.43'N, 85°0.248'W, 170 m: SMF 85940–41; Parque Nacional Patuca, Matamoros, 150 m: SMF 80824–25. **Nicaragua:** Bartola at Río San Juan, 10°58.37'N, 84°20.35'W, 30 m: SMF 82103; Parque Nacional Saslaya, 13°42.84'N, 84°58.66'W, 400 m: SMF 82875–77; Biosphere Reserve Bosawas, near Wiso, 13°59.67'N, 85°19.70'W, 246 m: SMF 78555; El Recreo, S side Río Mico, 25 m: KU 113016. **Panama:** Volante: SMF 89601; Cerro Tebata, Bocas del Toro Province: SMF 83638. **Surinam:** no further data: SMF 8374. **Trinidad:** Hotel Robinson Crusoe, Scarborough: SMF 65848; Alefounder, Grafton Estate: SMF 65849, 66185, 66204; Prospect Estate: SMF 66829. **Trinidad:** no further data: SMF 8376. **Venezuela:** Puerto Caballo: SMF 8375; between Guaramaco and San Fernando: SMF 8377.

*Thecadactylus solimoensis* – **Ecuador**: Pastaza Province, Arutam Field Station, 700 m east from the Camp (coordinates of the Camp): 1°47.28'S, 77°48.31'W, 790 m: SMF 91034.
